# Excited-state vibrational dynamics toward the polaron in methylammonium lead iodide perovskite

**DOI:** 10.1038/s41467-018-04946-7

**Published:** 2018-06-28

**Authors:** Myeongkee Park, Amanda J. Neukirch, Sebastian E. Reyes-Lillo, Minliang Lai, Scott R. Ellis, Daniel Dietze, Jeffrey B. Neaton, Peidong Yang, Sergei Tretiak, Richard A. Mathies

**Affiliations:** 1 Department of Chemistry, University of California, Berkeley, CA 94720 USA; 2 Department of Chemistry, Dong-A University, Busan, 49315 Republic of Korea; 3 Theoretical Physics and Chemistry of Materials, Los Alamos National Laboratory, Los Alamos, NM 87545 USA; 4 Departamento de Ciencias Físicas, Universidad Andres Bello, Santiago, 837-0136 Chile; 5 Department of Physics, University of California, Berkeley, CA 94720 USA; 6 Molecular Foundry, Lawrence Berkeley National Laboratory, Berkeley, CA 94720 USA; 7 Osram Opto Semiconductors GmbH, Leibnizstraße 4, Regensburg, 93055 Germany; 8 Kavli Energy NanoSciences Institute at Berkeley, Berkeley, CA 94720 USA; 9 Department of Materials Science and Engineering, University of California, Berkeley, CA 94720 USA; 10 Materials Sciences Division, Lawrence Berkeley National Laboratory, Berkeley, CA 94720 USA

## Abstract

Hybrid organic–inorganic perovskites have attractive optoelectronic properties including exceptional solar cell performance. The improved properties of perovskites have been attributed to polaronic effects involving stabilization of localized charge character by structural deformations and polarizations. Here we examine the Pb–I structural dynamics leading to polaron formation in methylammonium lead iodide perovskite by transient absorption, time-domain Raman spectroscopy, and density functional theory. Methylammonium lead iodide perovskite exhibits excited-state coherent nuclear wave packets oscillating at ~20, ~43, and ~75 cm^−1^ which involve skeletal bending, in-plane bending, and *c*-axis stretching of the I–Pb–I bonds, respectively. The amplitudes of these wave packet motions report on the magnitude of the excited-state structural changes, in particular, the formation of a bent and elongated octahedral PbI_6_^4−^ geometry. We have predicted the excited-state geometry and structural changes between the neutral and polaron states using a normal-mode projection method, which supports and rationalizes the experimental results. This study reveals the polaron formation via nuclear dynamics that may be important for efficient charge separation.

## Abstract (web summary)

Elucidating electron-phonon coupling in hybrid organic-inorganic perovskites may help us to understand the high photovoltaic efficiency. Here, the authors observe low-frequency Raman modes and related nuclear displacements of the Pb–I framework, indicating how these vibrational motions lead to polaron formation in perovskites.

## Introduction

Hybrid organic-inorganic perovskites (HOIP) composed of an organic cation and inorganic framework are one of the most promising new solar cell materials^1–10^. These materials are exciting because of their high power conversion efficiency (PCE) as well as their low-cost and facile fabrication^11^. Their efficiency has reached ~23% which is now comparable with single crystalline silicon-based solar cells^12^. However, understanding the underlying mechanisms of this high PCE is still a significant challenge.

Polycrystalline HOIP fabricated at low temperatures (~100 °C) has significant crystalline inhomogeneity: defects and grain boundaries typically introduce multiple energy-loss trap sites that may significantly lower the PCE^13–20^. This inhomogeneity is surprising because most other inorganic solar cell materials require pure crystalline composition for high PCEs^21,22^. Altogether these observations suggest that the inhomogeneity in the polycrystalline HOIP does not greatly influence the performance by protecting free charge carriers from ultrafast thermal relaxation, although these defects do accumulate in humidity-degraded materials^23–25^. Associated with the high PCE, polycrystalline HOIP exhibits a long lifetime (**τ**~1−3 μs) and a long diffusion length (*L*_D_**~**1−3 μm), as well as modest mobility (**μ**~30−100 cm^2^ V^−1^ S^−1^) for the free charge-carriers^26–28^. These characteristics indicate the efficient suppression of unfavorable ultrafast charge-recombination and suggest that unique excited-state dynamics may enable the longer charge-carrier lifetime and their impunity to thermal relaxation^29^.

The formation of a polaron, a charged quasi-particle, may account for the unique excited-state photo-physical properties in HOIP^30,31^. In principle, the charge-localized polaron state is energetically unstable without further stabilization. Once it is formed in a higher energy state, it should quickly relax via high energy phonon scattering^32^. However, polaron formation in HOIP can be favorable; electric dipole reorientation of the nearby organic cations not only minimizes the Gibbs free energy of the polaron but also protects the charges from other ultrafast thermal relaxations according to Zhu and coworkers^29,32,33^. They have successfully measured femtosecond time-resolved fluorescence of the hot electron–polaron in methylammonium lead iodide (MAPbI_3_) and shown that after a few hundred femtosecond relaxation, which is related to the methylammonium (MA) cation reorientation time, the polaron lives as long as ~100 ps^24^.

We have recently reported a theoretical prediction on the structure of the small electron polaron of MAPbI_3_, which exhibits not only MA dipole reorientations toward the charge but also the structural distortion of the inorganic Pb–I framework to stabilize the isolated charge^34^. In particular, the structural distortion associated with a polaron suggests that ground and excited-state potential energy surfaces (PES) have different energy minima along nuclear coordinates that are related to the distortion. Thus, coupled electron–phonon dynamics can be coherently excited on the polaron PES by impulsive excitation, where the associated reorganization energies (λ) and displacements (Δ) determine the polaron formation pathways^35,36^. These coherent dynamics have been detected by using femtosecond time-resolved spectroscopies^37,38^. For example, Wang et al.^39^ recently observed a single coherent wave packet oscillating at ~23 cm^−1^ in a MAPbI_3_ film using femtosecond transient absorption. However, additional experimental studies of excited-state dynamics in HOIP are necessary to resolve higher frequency vibrations disclosing the structural dynamics of polaron formation.

Herein, we report the production and analysis of coherent nuclear wave packets in MAPbI_3_ that oscillate at low frequencies ( < 100 cm^−1^) as measured by femtosecond impulsive stimulated-Raman spectroscopy (ISRS). We also present first principles density functional theory (DFT) simulations of the structural distortions associated with polaron state. The PbI_6_^4−^ octahedral charge-state geometry changes based on the experimental excited-state Raman displacements and the theoretical simulation are then compared. These results reveal coherent Pb–I vibrational normal mode dynamics that lead to the stabilized polaron state.

## Results

### Coherent wave packet measurement and analysis

**Fig. 1:**
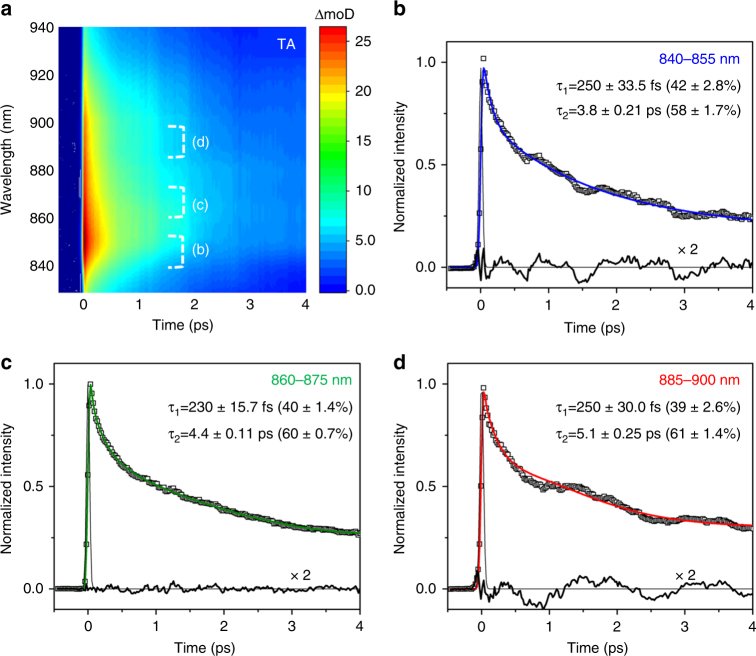
Transient excited state absorption spectrum shown in the 830–940 nm region of MAPbI_3_ perovskite excited by a ~40 fs FWHM pulse at 560 nm. **a** Contour plot of excited state absorption band centered at ~855 nm. **b**–**d** Time profiles measured at 840–855 (blue), 860–875 (green), and 885–900 nm (red) fit to the sum of two exponentials with the indicated constants. The residual profiles are also shown revealing underlying oscillations especially in the 840−855 and 885−900 nm regions

We probed the transient excited-state absorption (ESA) band of MAPbI_3_ perovskite in the near infrared (NIR) wavelength (830−940 nm) region which is distinct from the ground-state absorption (GSA), stimulated emission (SE) and other bleach bands (see Methods). Fig. 1a presents the contour plot of the ESA band centered at ~855 nm. There are no dynamic peak growths or shifts after the response-limited appearance; the ESA simply shows an exponential intensity decay in the observation window. Three representative time-profiles in the 840–855, 860–875, and 885–900 nm region are presented in Fig. 1b–d. The rise times are all resolution limited and each decay is well fit by a biexponential decay with constants of ~240 fs and 3.8–5.1 ps. Especially, the fits to the red and blue traces suggest that these data also contain underlying temporal oscillations. The difference residuals between the exponential fits and the experimental data in Fig. 1 confirm the existence of these temporal oscillatory components that are likely due to coherent nuclear wave packet dynamics.

**Fig. 2:**
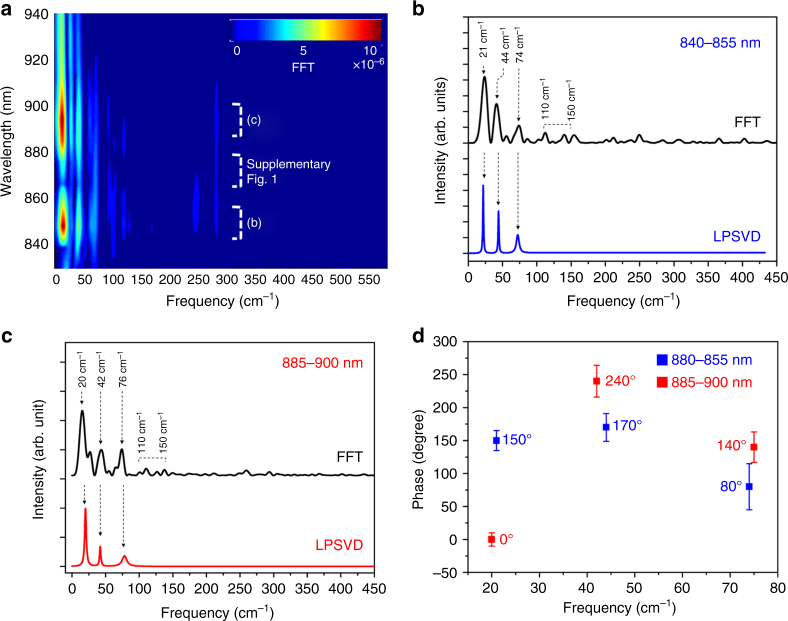
FFT spectra of Fig. 1. **a** Contour plot of FFT spectra measured in the entire probe pulse range for MAPbI_3_ perovskite (830–940 nm). **b**, **c** FFT and LPSVD spectra measured at 840–855 nm (blue) and at 885–900 nm (red). **d** Display of the relative phases of the three principal LPSVD peaks fit on the red and blue sides of the band

Fast Fourier transform (FFT) and linear prediction with singular value decomposition (LPSVD) analyses were performed to quantitate the oscillatory components in the NIR ESA band. The FFT spectra are presented from the residuals extracted from the exponential fittings at each wavelength (Fig. 2a), which mostly exhibit Raman modes lower than 150 cm^−1^. The FFT spectral intensities exhibit a bi-lobed pattern with strong components below 860 nm and above 880 nm but weak amplitudes around 865 nm near the ESA maximum (Supplementary Fig. 1). This result is consistent with the expectation that when a spectral band position is oscillating in time, the largest signal differences are on the red and blue sides, where the slope is highest and the lowest amplitude oscillations are near the peak center^40,41^. Figure 2b, c presents FFT and LPSVD results extracted in the indicated blue and red regions, which both exhibit common low frequency modes at ~20, ~43, and ~75 cm^−1^, supporting our wave packet analysis. In addition, the FFT spectra reveal very small 110 and 150 cm^−1^ Raman peaks which were also found in our previous resonance Raman studies^42^, but we were unable to perform a more detailed analysis of the latter bands here because of their weak intensities.

**Fig. 3:**
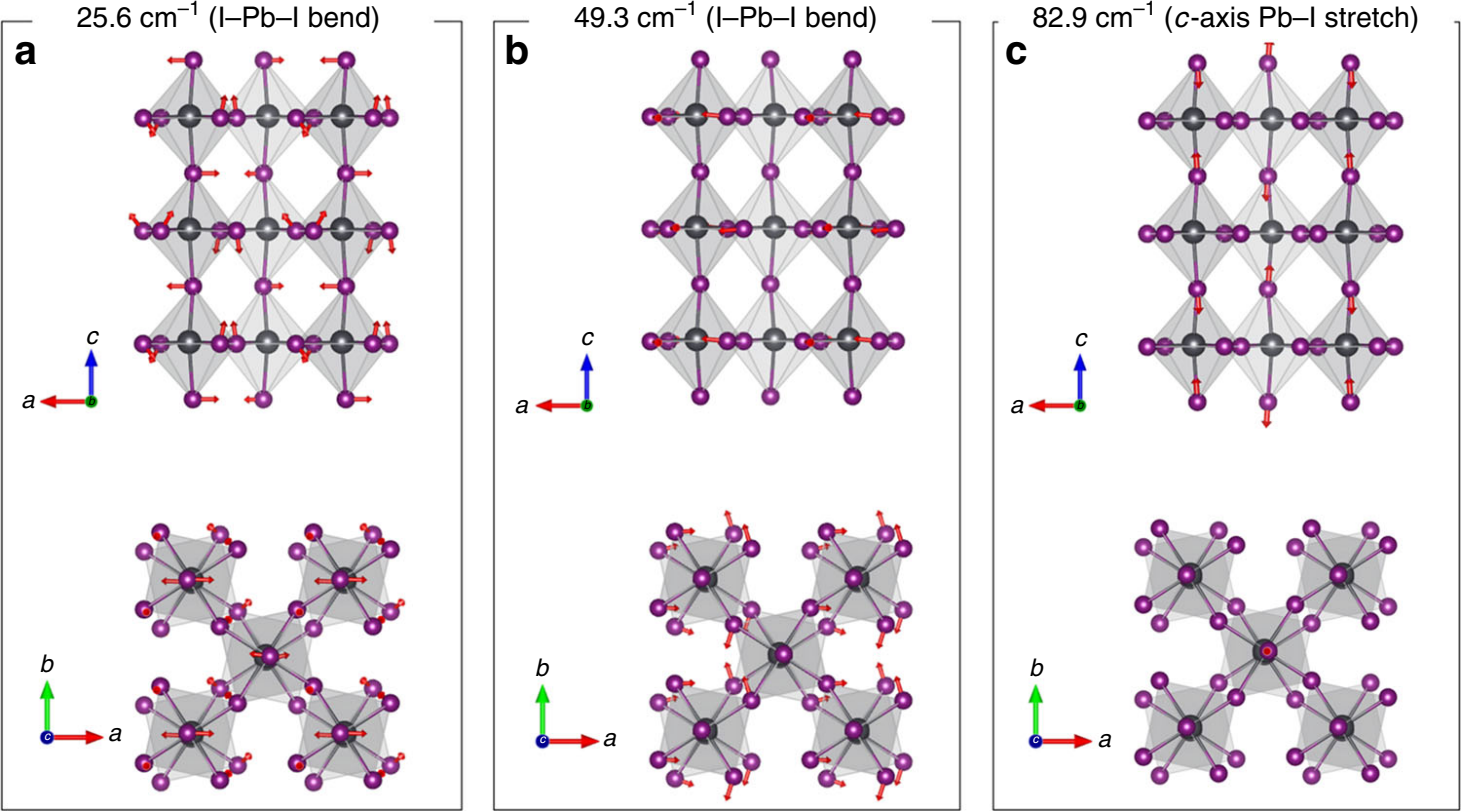
DFT calculated vibrational modes of MAPbI_3_. **a** Skeletal I–Pb–I bending motion of the PbI_6_^4−^ octahedron at 25.6 cm^−1^. **b** I–Pb–I bending motion on the *ab*-plane at 49.3 cm^−1^. **c** Pb–I *c*-axis stretching motion at 82.0 cm^−1^. Red arrows on the atoms indicate the direction and relative amplitude of displacements in the given vibrational modes

To examine whether these oscillatory signals are indeed due to band position oscillation of the ESA band we examined the relative phase of these oscillations. In principle, for the band position shift model they should be ~180° out of phase. However, it is sometimes difficult to extract the ideal phases and frequencies when there are highly damped and mixed oscillations with other frequencies. This ambiguity may result in serious overestimation using LPSVD analysis. In order to avoid this error, we cross-checked both the FFT and LPSVD results, as shown in Fig. 2. Figure 3d presents the phase parameters for the ~20, ~43, and ~75 cm^−1^ modes from the LPSVD results. The most intense wave packet at ~20 cm^−1^ has the largest phase shift from 150° to 0° which is nearly out of phase as expected. The 45 and 75 cm^−1^ modes exhibit relative phases of 240/170 and 140/80 on the red/blue sides that are roughly 60–70° out of phase. The deviation from the expected 180° phase differences might result from the somewhat larger errors in these phase determinations than for the 20 cm^−1^ mode. Especially, the ~180° out of phase features of the strongest ~20 cm^−1^ mode together with the amplitude pattern in Fig. 2a, support the idea that these signals are due to coherent nuclear wave packets which are propagating on the excited-state PES.

### Vibrational Raman mode calculation

To identify the vibrational modes associated with the observed wave packets, DFT calculations of vibrational normal modes in the room temperature tetragonal (*I4/mcm*) phase of MAPbI_3_ were performed using periodic boundary conditions (see Methods)^42^. Figure 3 presents the vibrational displacements (red arrows) calculated for the 25.6, 49.3, and 82.9 cm^−1^ modes of ground state MAPbI_3_ perovskite energetically lying in the region of interest. These vibrational modes are mostly composed of I−Pb−I bond bending and stretching motions unlike other higher frequency Raman modes above ~100 cm^−1^ which involve MA motion^42^. Figure 3a depicts a large I–Pb–I bending motion at 25.6 cm^−1^ which can be described as a B_1g_ mode in ${\mathrm{D}}_{4{\mathrm{h}}}^{18}$ symmetry group (*I4/mcm*) space group, while Fig. 3b presents another I–Pb–I bending motion in the ab-plane at 49.3 cm^−1^ (A_1g_ mode in ${\mathrm{D}}_{4{\mathrm{h}}}^{18}$). On the other hand, the 82.9 cm^−1^ mode in Fig. 3(c) displays the *c*-axis Pb–I stretching displacement (A_1g_ in ${\mathrm{D}}_{4{\mathrm{h}}}^{18}$) that effectively modulates the height of the octahedral cell. Based on the reasonable assumption that low frequency skeletal modes are similar in the ground and excited state, these three vibrational modes can be assigned to the experimentally measured wave packet modes at 21, 43, and 75 cm^−1^, respectively (Fig. 2) and used to depict distortion pathways of the octahedral PbI_6_^4−^ structure in the excited-state. Some disagreement between the DFT simulations and experiment is expected for low-frequency vibrational modes (here ~18, 9.0, and 9.0% for three modes in question) due to the shallow potential energy surface and the harmonic approximation used for analysis. For example, a recent study compared IR absorption and DFT results showing ~20% deviations in < 100 cm^−1^ region^43^. Thus, to reduce the discrepancy, we previously considered several additional effects that need to be taken into account to properly compare calculations with experiments which improved the reliability of our assignments^42^.

### Polaron state geometry calculation

**Fig. 4:**
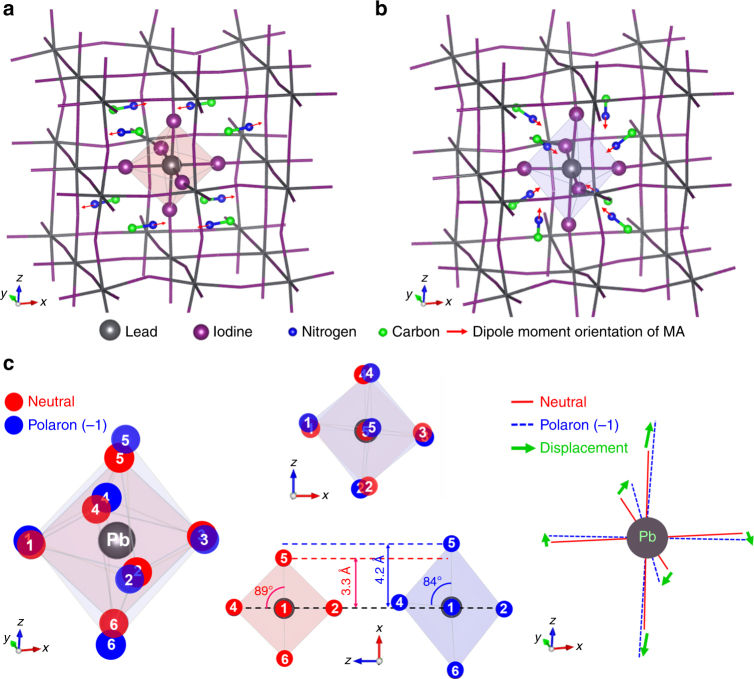
Theoretically predicted cluster structures of MAPbI_3_ perovskite. **a** Neutral cluster. **b** Polaron (−1) cluster. **c** Various perspective comparisons of the neutral (red sphere) and polaron (blue sphere) PbI_6_^4−^ octahedral structures centered in the clusters. The structural comparison shows that the polaron state geometry is more bent and elongated than the neutral, indicated by displacement vectors (green arrows)

To theoretically simulate the structural changes of excited PbI_6_^4−^ DFT simulations of finite clusters of MAPbI_3_ perovskites were performed following procedures previously outlined in our recent work^34^. The subsequent analysis is based on CAM-B3LYP functional (see our previous work^34^ and Methods). Specifically, we obtained geometries of a MAPbI_3_ neutral cluster and a cluster with an extra electron mimicking negative polaron generation. The computationally determined ground state structure was previously verified by XRD experiments, and is therefore more reliable compared to the geometry of charged states^34^. Figure 4 shows the structurally optimized clusters of the simulated neutral and negative polaron states of MAPbI_3_ perovskite. In the polaron structure, the methylammonium counterions reorient their electric dipoles (indicated by red arrows) toward the center Pb atom to minimize the free energy of the localized electron density in the center Pb atom^34^. In addition, the Pb–I bonds, especially in the central PbI_6_^4−^ octahedra, also experience substantial structural changes, when an electron is added. Figure 4c compares the central PbI_6_^4−^ octahedron structure of the neutral (**S**_neu_, red sphere) and the polaron (**S**_pol_, blue sphere). The most unique structural difference between **S**_neu_ and **S**_pol_ is the octahedron height along *z*-axis; the polaron PbI_6_^4−^ has a Pb–I(5) distance of 4.2 Å which is considerable longer than 3.3 Å for the neutral one. In addition, the neutral PbI_6_^4−^ has a nearly right angle (89°) for the I(5)–Pb–I(4) bond, while in the polaron the bond angle is 84°. Thus, the polaron has a distorted and bent octahedral structure, while the neutral geometry forms a nearly regular octahedron. These differences are indicated by the displacement vectors (green arrows) in Fig. 4c, which are composed of bending and stretching motions of the I–Pb–I bond.

The structural differences predicted for the excited polaron state geometry should be related to the vibrational relaxation toward the charge state minimum that occurs after Franck–Condon (FC) excitation of the neutral form. Thus, the measured vibrational modes oscillating in the ESA should be the modes that trigger the nuclear reorganization that forms the excited polaron state. In order to test this hypothesis, we quantitatively investigated the relation between the experimental excited-state vibrational oscillation intensities (Figs. 1–3) and the polaron structural predictions (Fig. 4).

### Experimental and theoretical displacement analysis

**Fig. 5:**
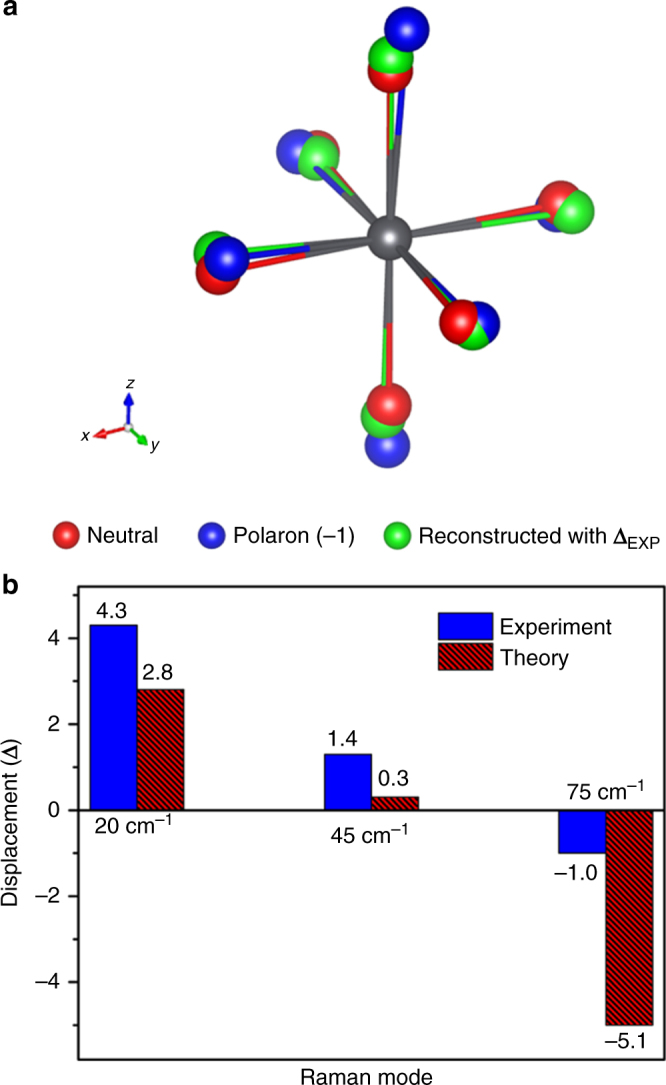
Structural comparisons between experiment and theory. **a** Reconstructed PbI_6_^4−^ octahedral structure (green) from **∆**_Exp_ and Eq. (1) compared with the theoretically predicted neutral (red) and polaron (blue) state structures. **b** Displacement comparisons between the experiment (**∆**_Exp_) and theory (**∆**_Theo_)

The key part of our analysis is relating the oscillatory signals in the ESA in Figs. 1 and 2 with the displacement of the excited state of perovskite along the observed vibrational modes. In the time-dependent picture of Raman intensities, these excited state oscillations are precisely the nuclear motions that give rise to the resonance Raman scattering process. Since the Raman scattering cross-sections (**σ**_R_) are approximately proportional to ∆^2^*ω*^2^ in the short-time limit, where ∆ and *ω* are the ground-to-excited state displacement of the potential minima and the vibration frequency, respectively^44–46^, the relative displacement ratio for each Raman peak can be determined. Using this relationship, the relative displacement ratio (**∆**_Exp_) is found to be ±4.3, ±1.4, and ±1.0 for the ~20, ~45, and ~75 cm^−1^ modes in the impulsive stimulated Raman spectra (ISRS) in Fig. 2. The absolute phases cannot be determined from the experimental data alone because of the **∆**^2^ relationship. The **∆**_Exp_ result shows that the I–Pb–I bending motion at ~20 cm^−1^ is the most highly displaced vibrational normal mode, and thus, the most responsible for the excited-state PES deformation that we have probed in the TA. The other bending and stretching modes provide lower displacement contributions to the excited-state PES. Thus, using the ∆_Exp_, we can experimentally estimate a polaron state structure (**S**_Exp_) given by

1 $${\mathbf{S}}_{{\mathrm{Exp}}} = {\mathbf{S}}_{{\mathrm{neu}}} + p({\mathrm{\Delta }}_{{\mathrm{Exp}}} \cdot {\mathbf{c}}_{{\mathrm{neu}}})$$

where *p* is a proportional factor and ${\mathbf{C}}_{{\mathrm{neu}}}$ is a matrix containing the Cartesian-coordinate vibration eigenvectors of each atom of the neutral state. Notably, Eq. (1) does not rely on the less accurate theoretical **S**_pol_ for excited state structure. Figure 5a presents the comparison of **S**_neu_ (red), **S**_pol_ (blue), and **S**_Exp_ (green) with *p* *=* 1.6 from the best nonlinear square fit toward **S**_pol_ (${\boldsymbol{\chi }}_r^2$~1.43) when 4.3, 1.4, and −1.0 are used for ${{\Delta }}_{{\mathrm{Exp}}}$. We determined the phases of the ${{\Delta }}_{{\mathrm{Exp}}}$ from inspection of the theoretical polaron geometry in Fig. 4b. It is evident that the green atoms of the reconstructed **S**_Exp_ move toward the blue (**S**_pol_) from the red (**S**_neu_) locations, thus affirming the successful simulation of the theoretically observed polaron state structure using the relative displacement ratio, **∆**_Exp_.

A complementary approach for analyzing these geometric changes is to perform a normal mode projection from the **S**_neu_ to **S**_pol_ by relying solely on theoretical predictions^47^. The theoretical displacement (∆_Theo_) is given by

2 $$\Delta _{{\mathrm{Theo}}} = {\mathbf{l}}_{{\mathrm{neu}}}^{ - 1}{\mathbf{c}}_{{\mathrm{neu}}}^T\left( {{\mathbf{S}}_{{\mathrm{pol}}} - {\mathbf{S}}_{{\mathrm{neu}}}} \right),$$

where **l** is a diagonal matrix containing $l_{ii} = \left( {\frac{\hbar }{{2\pi \nu _i}}} \right)^{\frac{1}{2}}$, and $\nu _i$ is the *i*^th^ vibration frequency. Δ_Theo_ are determined to be +2.8, +0.3, and −5.1 for the 20, 45, and 75 cm^−1^ vibration modes, respectively. In addition, the excited-state reorganization energy (λ) is calculated to be 145 meV (~1170 cm^−1^) given by ${\mathit {\lambda }} = \frac{h}{2}{\sum} {\nu _i{{\Delta }}_i^2}$, where *h* is Planck constant. This value is very consistent with the band gap stabilization energy (~160 meV) found in a periodic boundary condition (PBC) DFT calculation^34^, indicating that our observed vibration modes represent the majority of polaron stabilization dynamics.

Figure 5 shows that the combined ∆_Exp_ and ∆_Theo_ results are in good qualitative agreement; both experimental and theoretical displacements show that the 20 cm^−1^ displacement is larger than the 45 cm^−1^ displacement, both confirming that the whole octahedral I–Pb–I bending motion at ~20 cm^−1^ provides strong displacements toward the polaron state. However, the displacement −5.1 of 75 cm^−1^ mode of Δ_Theo_ is larger than the experimentally observed Δ_Exp_ at 75 cm^−1^. This inconsistency principally results from the different heights of **S**_pol_ (4.2 Å) and **S**_Exp_ (3.6 Å) which result from an overestimation of **S**_pol_ due to the use of limited atom numbers in the calculation and an underestimation of **S**_exp_ due to the limited time-resolution of the experiment which damps the oscillatory signal strength for the higher frequency mode. However, both our methods (Eqs. (1) and (2)) present a consistent picture about the elevated octahedral heights from **S**_neu_. Thus, the theoretically predicted polaron geometry is nicely supported by the experimentally resolved vibrational displacements, showing that I–Pb–I bending and stretching motions are the key displacements stabilizing polaron formation.

## Discussion

Our nuclear wave packet results and theoretical simulations on MAPbI_3_ perovskite show how the polaron state is coherently generated on the displaced PES. Electronic excitation above the band gap (~1.65 eV) of MAPbI_3_ produces the coherences as determined in the current study, ultimately leading to the separation of an initial excitation into free charges in view of weak excitonic effects in MAPbI_3_^48,49^. The observed dynamic vibrational excitations facilitate this relaxation toward different structures, where a localized charge is further stabilized through the structural deformation of the Pb–I framework (Fig. 4) and the MA electric dipole reorientation; this is the polaron state hypothesis^34^. The reorientation time (τ) of the solvated MA was measured to be ~150 fs by using optical Kerr-gate spectroscopy and ultrafast IR spectroscopy; this time is most relevant for ultrafast coherent solvation in solutions^33,50^. In this context, we have found small FFT peaks at ~150 cm^−1^ (see Figs. 1 and 2) which are likely due to MA’s libration motion^42^. However, the MA coherence is found to be much smaller than the Pb–I coherences, showing that the Pb–I motions present the principal FFT peaks at low frequencies. This suggests that the MA reorientational motion is likely to be a more continuous (incoherent) solvent-like movement stabilizing the localized polaron^50^, while the measured Pb–I vibrations are coherent processes which trigger the Jahn–Teller distortion.

Pb and I atoms are covalently bonded to form the sturdy perovskite framework. However, once more charge character is introduced (especially, into the Pb atom), this Pb–I framework becomes more ionic^51^ and the transition is facilitated by Pb–I structural distortions shown as the bending and elongations of the Pb–I bonds mirrored in our theoretical studies (Fig. 4). Our key finding is that this distortion is effectively performed by Pb–I coherent vibrational motions over a few picosecond time scale. Interestingly, both experiment and theory agree that the 21 cm^−1^ mode (I–Pb–I bending) should be the principal nuclear distortion motion for polaron formation, in good agreement with the strong electron–phonon coupling at 0.65 THz (22 cm^−1^) probed by femtosecond terahertz emission^51^ and the light-induced I–Pb–I angle displacement measured by electron scattering^52^.

**Fig. 6:**
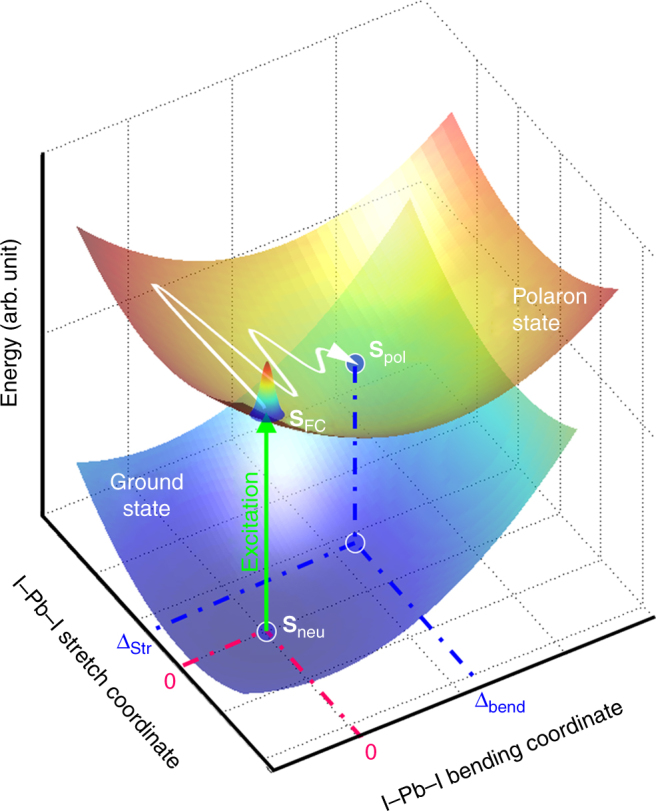
Excited-state potential energy surface diagram. The diagram displays the atomically displaced (**∆**_Str_, **∆**_Bend_) polaron state surface relative to the ground state (0, 0) neutral surface after excitation. A coherent nuclear wave packet oscillates along the I–Pb–I stretching coordinate and propagates toward **∆**_Bend_ along the I–Pb–I bending coordinate. **S**_FC_, **S**_neu_, and **S**_pol_ are relative structures of Franck–Condon state, neutral, and polaron states

Figure 6 displays a schematic two-dimensional PES diagram for the polaron state formation. The PES for the neutral and polaron states have different potential minima which are **S**_neu_(**0**, **0**) and **S**_pol_(**Δ**_Str_, **Δ**_Bend_), respectively. The geometry changes are determined predominantly by an I–Pb–I bending normal mode at 21 cm^−1^ (Fig. 3a) and an I–Pb–I *c*-axis stretching normal mode at 75 cm^−1^ (Fig. 3c). After excitation to the Franck–Condon state, **S**_FC_(**0**, **0**), the displacements cause the excited-state nuclear wave packets to evolve along these multi-dimensional I–Pb–I vibration coordinates coherently propagating excited-state population toward the polaron state minima **S**_pol_(**Δ**_Str_, **Δ**_Bend_) in the first few picoseconds. Thus, we suggest that coherent polaron formation dynamics is linked to the efficient charge-separation of MAPbI_3_ perovskite by directing the phase–space evolution to the polaron state faster than competing relaxation mechanisms. Other reports have suggested the subsequent dynamics of charge carriers as large polarons, where limited charge–carrier mobility was linked to coupling to LO phonon branches with frequency of 88 cm^−1^ ^53,54^.

In summary, we performed femtosecond TA and DFT simulations to probe the excited-state dynamics of MAPbI_3_ and found evidence for coherent skeletal vibration dynamics that lead to formation of a polaron state underscoring charge separation. The low frequency wave packet modes are observed principally for the Pb–I bending and stretching vibrations resulting from the different geometry of the polaron state compared to the neutral state. We analyzed the experimental and theoretical displacements and confirmed that the theoretical negative polaron state structure is supported by the experimental Raman intensity analysis results. Thus, the distorted octahedral geometry of the PbI_6_^4−^polaron state structure is characterized, and its initial formation dynamics are demonstrated to be dominated by the coherent nuclear wave packet motion. The high efficiency of MAPbI_3_ perovskite solar cells may be yet another example of the importance of vibrational coherence in efficient photochemical dynamics^55–57^.

## Methods

### Sample preparation

Samples were prepared by dissolving 0.76 mmol PbI_2_ (99.99%, Sigma-Aldrich) and 2.28 mmol CH_3_NH_3_I (Dyesol Inc.) in 1 mL anhydrous dimethylformamide (DMF, Sigma-Aldrich), and stirred overnight. The prepared solution was spun on O_2_-plasma treated glass substrates at 1500 rpm for 60 s, and then annealed at 120 °C for 60 min in a N_2_-filled glovebox. Poly(methyl methacrylate) (PMMA) was coated on the fresh MAPbI_3_ layer to protect it from the atmosphere. We have checked powder X-ray diffraction (XRD) measured by using a Bruker AXS D8 Advance diffractometer with a Cu Kα source. (Supplementary Fig. 2)

### Pump-probe spectroscopy

A home-built Kerr lens mode-locked Ti:sapphire oscillator (~20 fs FWHM, 5.3 nJ/pulse, 91 MHz) seeded a Ti:sapphire regenerative amplifier (B.M. Industries, Alpha 1000 US, 991 Hz, 70 fs, 780 μJ/pulse, λmax = 790 nm) pumped by a Q-switched Nd:YLF (B.M. Industries, 621-D). The fundamental at 790 nm was used to generate visible pump pule (~35 fs FWHM, 150 nJ/pulse, λmax=560 nm) by a non-collinear optical parametric amplifier (NOPA). A negatively chirped mirror pair (Layertec GmbH) was used to compress the pump pulse. A small portion of the fundamental is focused onto a 3 mm thick sapphire plate to generate a broadband continuum probe pulse (~10 fs FWHM, ~3 nJ/pulse). The probe pulse was temporally compressed in the 830–940 nm region with a pair of BK7 dispersion prisms (Supplementary Fig. 3). The cross-correlation function was measured to be a Gaussian function with ~44 fs FWHM by using the optical Kerr effect in cyclohexane (Supplementary Fig. 4). The power of the pump pulse was reduced to ~6 nJ to avoid thermal and photochemical damage to the sample. A custom-made N_2_ gas-flow chamber was used to prevent humidity-degradation of the MAPbI_3_ sample. A monochromator (Instruments SA, HR320), a laser-synchronized CCD (Princeton Instruments, PIXIS), and an optical chopper (Newport, 3501) were used to measure the optical density (OD) differences in every pump/probe cycle (Supplementary Fig. 3). Python and Matlab software were used to control the experimental instruments and analyze data, respectively.

### Calculation of vibration frequencies

First-principles density functional theory (DFT) calculations were performed using VASP^58,59^. Our VASP calculations use the generalized gradient approximation (GGA)^59^, and projected augmented-wave pseudopotentials^60,61^. Structural and electronic properties were computed using a plane-wave energy cut-off of 500 eV and a 4 × 4 × 4 Monkhorst–Pack k-point grid^62^, including spin–orbit interactions (SOI). Lattice constants and internal atomic positions were relaxed starting from the experimental structure, without symmetry constrains, until forces were smaller than 1 meV/Å. Phonon eigenfrequencies and eigenvectors were calculated in the harmonic approximation, neglecting SOI. Our phonon calculations use density functional perturbation theory and a finer 6 × 6 × 6 k-point grid. Selected phonon modes and electronic orbitals were plotted using VESTA. The room temperature tetragonal (I4/mcm) phase of (CH_3_NH_3_)PbI_3_ is simulated in a $\sqrt 2$ × $\sqrt 2$ × $2$ perovskite unit cell. The tetragonal structure consists of a PbI_6_ octahedral rotation around the *z*-axis (a^0^a^0^c^−^ in Glazer notation) and an antiparallel orientation of the CH_3_NH_3_ (MA) molecules^42,63,64^.

### Optimization of polaron structure

All calculations of HOIP isolated clusters were performed using the CAM-B3LYP functional combined with the LANL2dz (for Pb and I) and 6-31 G* (for N, C, and H) basis sets using Gaussian 09 software package^65^. In addition, we include a polar solvent (ε = 78) via the conductor-like polarizable continuum model^66^.

Several considerations were taken into account when designing the isolated HOIP structures. We started with the low temperature cubic lattice parameters that were derived from variable temperature powder x-ray diffraction experiments^67^. In order to minimize dangling bonds, the cluster was terminated on all sides with MAI. Equations 1 and 2 ensure that the system is overall neutral

1 $$6n_{\rm Pb} = 2n_{I_1} + n_{I_2}$$

2 $$2n_{\rm Pb} + n_{\rm MA} = n_{I_1} + n_{I_2}$$

Here $n_{\rm Pb},\;n_{\rm {MA}},\;{\rm and}\;{\it n}_{\rm I}$ are the number of Pb, MA, and I atoms, respectively. *I*_1_ corresponds to an atom on the surface and is only bonded to one Pb atom, while *I*_2_ corresponds to an I atom that is bonded to two Pb atoms. The cluster needs to be large enough to include at least 8 MA cations surrounding the central Pb atom. To fulfill all conditions, we end up with a cluster having the following stoichiometry: MA_54_Pb_27_I_108_ (*I*_1_=*I*_2_=54). A cube fully terminated by MA and I would require 64 MA molecules. In order to realize charge balance between the ions and maximize symmetry, MA’s were removed from the 8 corners and on two opposite edges.

In addition, the polaron geometry in HSE hybrid potential was checked. Supplementary Fig. 5 shows the optimized geometries from CAM-B3LYP (19 to 60% HF) and HSE (25 to 0% HF) functionals. The arrows point in the direction the atoms move in the formation of the polaron. This shows that the trend (I–Pb–I bending & *c*-axis elongation) still holds although the amplitude changes slightly.

### Raman mode assignment

Since we measured the excited-state Raman signals originated from the structural displacements (**Δ**) between the ground and excited states in MAPbI_3_, we considered Rashba band splitting and polaron formation for the reason of the excited-state displacements. The former one is related to IR-active vibration modes, because the splitting is promoted with directional structural strains. Meanwhile the latter, the polaronic PbI_6_^4−^ is rather induced by symmetric structural changes from the neutral PbI_6_^4−^ of the ground state, because a negative charge (−1) is localized in 6p orbital of the center Pb. Thus, this polaronic structure shows the Pb-centered symmetric distortion shown in Fig. 4. Based on this idea, our Raman mode assignments have been done with the octahedral PbI_6_^4−^ Raman modes in the D_4h_ Raman mode pool^43^. Thus, the Pb-centered Raman modes are easily discriminated from other IR-active and Pb-moving Raman modes (Supplementary Fig. 6).

### Data availability

All data supporting the findings of this study are available from the corresponding author upon reasonable request.

## Electronic supplementary material

Supplementary Information
