# Supplementary material for: Excited-state vibrational dynamics toward the polaron in methylammonium lead iodide perovskite
Source: Nat Commun. 2018 Jun 28;9:2525. doi: 10.1038/s41467-018-04946-7 (PMC6023914; doi:10.1038/s41467-018-04946-7)
Supplement: Supplementary file 1 — Supplementary Information [file 41467_2018_4946_MOESM1_ESM.pdf]

<u>Supplementary Information</u>

Excited-State Vibrational Dynamics Toward the Polaron in Methylammonium Lead Iodide Perovskite

Myeongkee Park,[1,2] Amanda J. Neukirch,[3] Sebastian E. Reyes-Lillo,[4,5,6] Minliang Lai,[1] Scott R. Ellis,[1] Daniel Dietze,[7] Jeffrey B. Neaton,[5,6,8] Peidong Yang,[1,8,9,10], Sergei Tretiak,[3] and Richard A. Mathies[1,*]

[1] Department of Chemistry, University of California, Berkeley, California 94720, United States

[2] Department of Chemistry, Dong-A University, Busan, 49315, Republic of Korea

[3] Theoretical Physics and Chemistry of Materials, Los Alamos National Laboratory, Los Alamos, New Mexico 87545, United States

[4] Departamento de Ciencias Físicas, Universidad Andres Bello, Santiago 837-0136, Chile

[5] Department of Physics, University of California, Berkeley, California 94720. United States

[6] Molecular Foundry, Lawrence Berkeley National Laboratory, Berkeley, California 94720, United States

[7] Osram Opto Semiconductors GmbH, Leibnizstraße 4, Regensburg 93055, Germany

[8] Kavli Energy NanoSciences Institute at Berkeley, Berkeley, California 94720, United States

[9] Department of Materials Science and Engineering, University of California, Berkeley, California 94720, United States

[10] Materials Sciences Division, Lawrence Berkeley National Laboratory, Berkeley, California 94720, United States

*ramathies@berkeley.edu

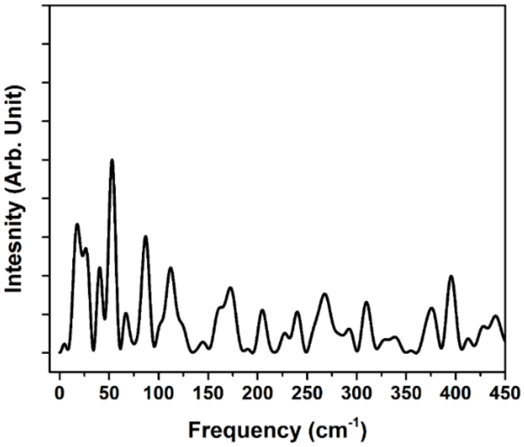

Supplementary Figure 1. FFT result measured at 860–875 nm.

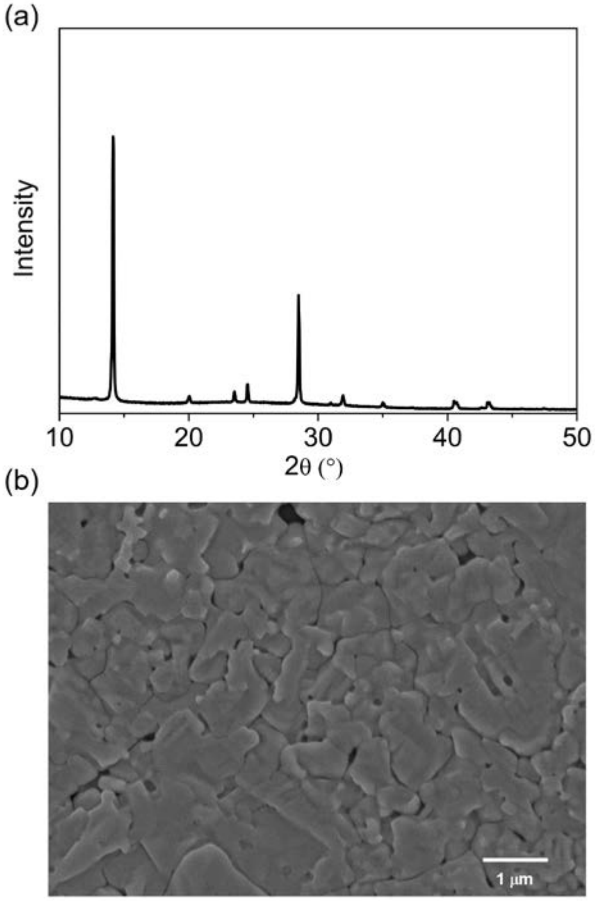

Supplementary Figure 2. (a) X-ray diffraction spectrum and (b) SEM image of the polycrystalline

$MAPbI_3$ film. No contribution from PbI_2 is at 2θ=12.64° was observed.

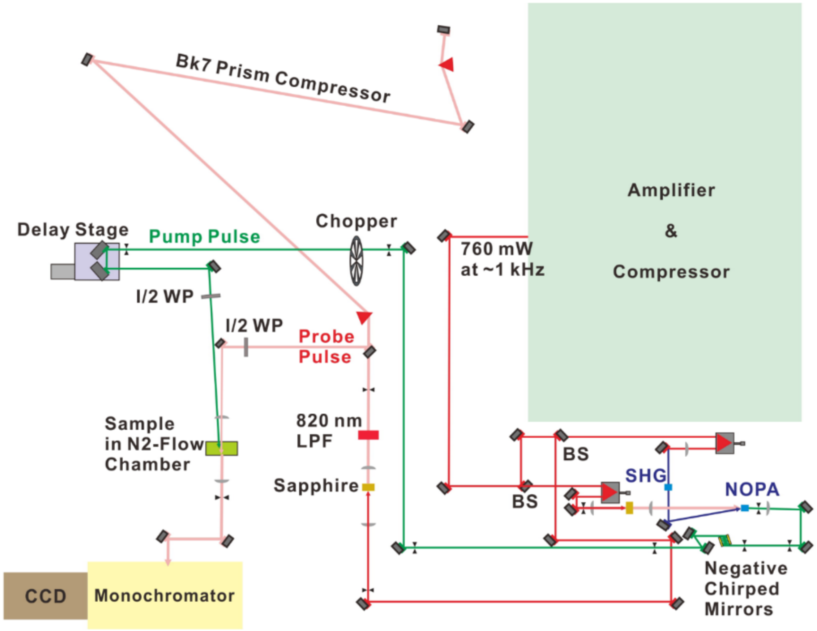

$$\Delta \mathrm{OD} = -\log \frac{I_{\text{probe, pump pulse on}}}{I_{\text{probe, pump pulse off}}}$$

Supplementary Figure 3. Diagram of femtosecond pump-probe spectroscopy apparatus. BS: Beam splitter, SHG: Second-harmonic generation, NOPA: Non-collinear optical parametric amplifier, LPF: Long-pass filter, and WP: λ/2 wave plate.

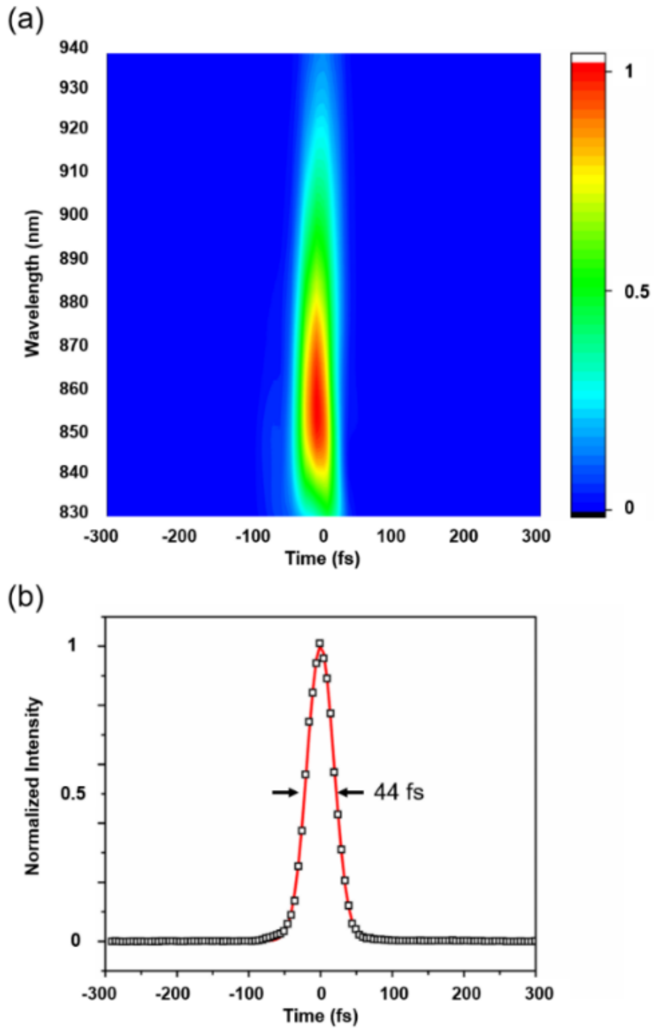

Supplementary Figure 4. (a) Cross-correlation spectrum between pump and probe pulses and (b) temporal profile measured at 850 nm fit to 44 fs FWHM Gaussian function.

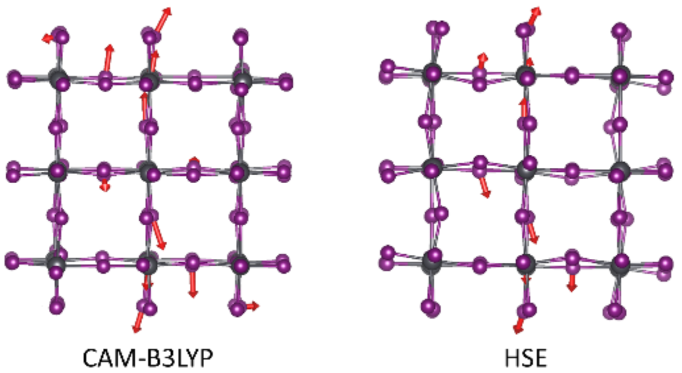

Supplementary Figure 5. Optimized polaron structure by using CAM-B3LYP and HSE hybrid potentials. The arrows indicate the magnitude and direction the atoms travel when the polaron forms.

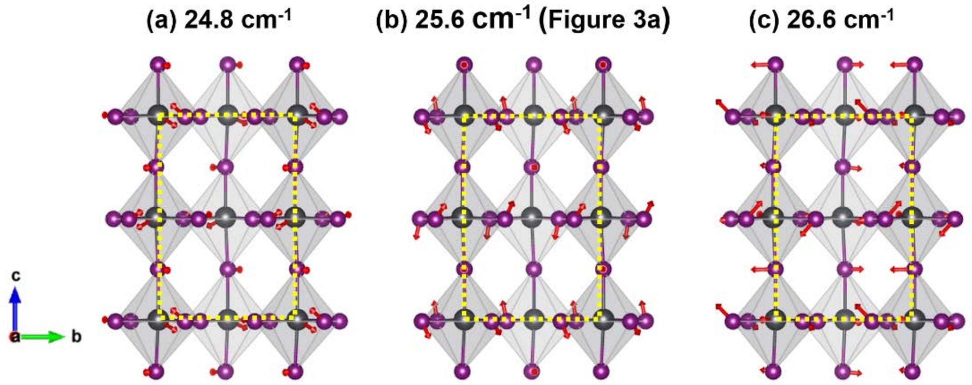

Supplementary Figure 6. Calculated Raman-active modes at similar frequency (~25 cm^{-1}). The yellow dashed line indicates the unit cell dimension. The PbI_6^{4-} motion at (b) 25.6 cm^{-1} only shows the octahedral Pb-centered motion giving symmetric motion, while the others include the motion of Pb.
